# Enzymatic Reactions Dictated by the 2D Membrane Environment

**DOI:** 10.1021/acs.jpclett.5c00988

**Published:** 2025-06-24

**Authors:** Ru-Hsuan Bai, Chun-Chen Lin, Chun-Wei Lin

**Affiliations:** Department of Chemistry, 34881 National Tsing Hua University, Hsinchu, Taiwan 300044

## Abstract

The cell membrane is a critical component of cellular architecture, serving not only as a physical barrier enclosing the cytosol but also as a dynamic platform for various biochemical reactions. Due to the unique two-dimensional and fluidic environment of the membrane, reactions that occur on its surface are subject to specific physical constraints. While membrane-mediated reactions are known to play key roles in cellular regulation, their advantages and limitations remain inadequately explored. In this study, we reconstitute a classic proteolytic cleavage reaction at the membrane interface, designed for the real-time kinetic analysis down to the single-molecule level. By systematically altering the enzyme-membrane affinity, we examined enzyme–substrate interactions under various conditions. Our findings reveal that while the membrane environment significantly enhances enzymatic turnover rate, it also imposes diffusion limitations that immediately reduce this turnover rate over time. By adjusting the enzyme’s membrane affinity to an intermediate level, we enable the enzyme to “hop” on the membrane surface, overcoming these diffusion constraints and sustaining high enzymatic turnover rate with faster kinetics. These results highlight the dual role of the membrane environment in regulating biochemical reactions, balancing enhanced reactivity with physical limitations. Moreover, the ability to dynamically tune membrane affinity to optimize reactions underscores the cell’s capacity to regulate enzymatic processes efficiently. This study provides critical insights into the role of the cell membrane in biochemical reactions and offers a broadly applicable framework for understanding membrane-associated interactions in biological systems.

## Graphical abstract

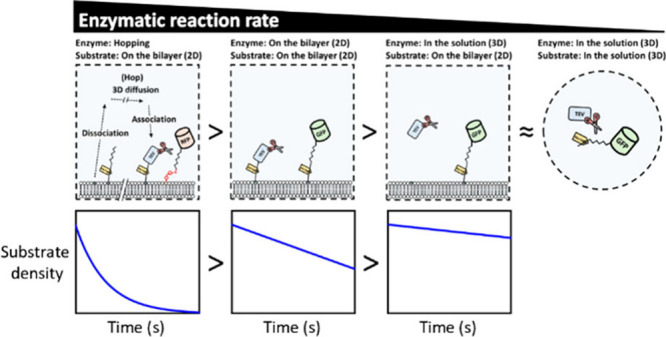

The plasma membrane is a key component of the cell, acting as a physical barrier that separates the intracellular environment from the extracellular space. It supports two distinct reaction systems of different dimensionalities: the three-dimensional (3D) compartments within the cell and the two-dimensional (2D) interface provided by the membrane itself. Beyond serving as a boundary, the membrane plays an active role as a 2D platform for diverse cellular processes, particularly those involved in signal transduction and intercellular communication.

In addition to membrane proteins, many biomolecules in the vicinity of the membrane directly or indirectly contribute to essential processes such as signal transduction, molecular transport, and cell–cell recognition.^1–7^ Signal transduction pathways, in particular, often involve not only membrane-bound proteins but also cytoplasmic proteins that are actively recruited to the membrane surface. This raises an important biological question: why does the cell localize multiple downstream signaling components to the membrane instead of allowing signal propagation to proceed entirely in the cytosol?

A classical example is the receptor tyrosine kinase epidermal growth factor receptor (EGFR), which resides at the plasma membrane and regulates cellular proliferation and survival through the mitogen-activated protein kinase (MAPK) pathway. Ligand binding, such as by epidermal growth factor (EGF),^8^ induces EGFR dimerization and autophosphorylation of tyrosine residues in its cytoplasmic domain.^9–13^ This, in turn, facilitates the recruitment of the adaptor protein Grb2 and the guanine nucleotide exchange factor SOS, leading to the activation of the membrane-associated Ras protein.^14^

A similar mechanism is observed with G-protein coupled receptors (GPCRs), which also form signaling complexes at the membrane. Upon ligand activation, GPCRs recruit heterotrimeric G proteins—composed of α, β, and γ subunits^15–20^ —to form agonist-bound GPCR–G protein complexes.^21^ These examples underscore the membrane’s role as a central hub for the assembly of signaling complexes involving ligands, receptors, and downstream effectors.^22,23^

The 2D nature of the membrane provides several key advantages for signaling. These include an increase in local concentration of signaling molecules upon recruitment,^2,14,24^ spatial confinement that limits diffusion and enhances reactivity,^25^ the formation of protein condensates that function as reaction centers,^26–30^ and the ability to modulate the conformational energy landscapes of membrane proteins to drive specific reactions.^31^ Taken together, the plasma membrane is not merely a structural boundary, but a dynamic and essential platform for orchestrating cellular signaling and other critical biological activities.

The advantages of two-dimensional (2D) systems for biochemical reactions in cells have been discussed for decades. Adam and Delbrück were among the first to propose that dimensionality reduction can accelerate biological reactions by enhancing the association between membrane-bound species and their binding partners.^32^ More recently, both theoretical and experimental studies have supported the idea that membrane localization can promote molecular association.^33,34^

When two proteins are membrane-bound, their lateral movement becomes restricted, and their rotational degrees of freedom are also reduced. As a result, the entropic cost of their interaction is significantly lower compared to that of two proteins freely diffusing in three-dimensional (3D) solution.^2,25^ Pólya’s theorem further demonstrates that a molecule undergoing a random walk on a 2D surface has a higher probability of encountering another molecule than in 3D space.^35^ These principles highlight the intrinsic advantages of membrane association in enhancing protein–protein interactions.

However, membrane localization is not universally beneficial. When a cytosolic enzyme binds to the membrane, its diffusion is typically slowed by nearly 2 orders of magnitude. This reduced mobility decreases the encounter rate between proteins by approximately 3- to 30-fold.^2^ Consequently, reactions between membrane-anchored proteins can become diffusion-limited. Despite this, the 2D confinement of molecules dramatically increases their local concentration—by more than 600-fold in some cases^2,25,36^ —enabling interactions that might be improbable in solution.^37^

In addition to reducing degrees of freedom, the membrane’s heterogeneous catalytic surface can further enhance reactivity via surface confinement effects. On heterogeneous surfaces, effective concentrations can be substantially increased.^38,39^ This concept has been extended beyond in vivo membranes to in vitro systems, where soluble enzymes are immobilized on solid supports or model bilayers. Such confinement not only improves enzyme stability^40–44^ but also enhances their regio- and stereoselectivity.^45–47^

Spatial organization is also critical in more complex systems involving enzymatic cascades. The enzymatic turnover rate in these systems is often influenced by the average distance between enzymes, underscoring the importance of spatial control.^41,48–50^ Moreover, membrane composition modulates the local environment and physical properties, which in turn affect surface reaction kinetics. For example, cholesterol accumulation slows lipid lateral diffusion^51^ and can reduce kinase turnover rates by approximately 3.5-fold when 1% cholesterol is present in the bilayer.^52^ Similarly, the intrinsic curvature of lipids—dictated by the shape of their headgroups and tails—can promote membrane curvature and enhance protein interactions.^53,54^

Although the increase in local concentration due to dimensionality reduction generally outweighs the reduction in mobility on the membrane,^14,25,55^ the low diffusion rate can cause substrate depletion around membrane-bound enzymes. This makes such reactions more prone to becoming diffusion-limited, especially when substrate replenishment is restricted by slow lateral diffusion. Therefore, the kinetics of membrane-associated reactions reflect a complex balance of advantages and limitations inherent to the 2D membrane environment. Beyond molecular encounter rates, many other factors influencing reaction kinetics must be experimentally interrogated. Ultimately, a full understanding of biochemical reactions requires studying them directly on membrane surfaces under biologically relevant conditions.

In this work, we systematically investigate a biological reaction mediated by the two-dimensional (2D) membrane environment using the Tobacco Etch Virus (TEV) proteolytic cleavage as a model system. We anchored the substrate of TEV protease onto supported lipid bilayers (SLBs) and examined the reaction kinetics when TEV protease is recruited to the membrane.

To track the kinetics of the cleavage reaction, we fused enhanced green fluorescent protein (eGFP) to the TEV substrate as a fluorescent probe. The eGFP fluorescence at the membrane surface was monitored in real time using total internal reflection fluorescence (TIRF) microscopy. Fluorescence intensity was then converted into absolute molecular counts through a series of precise calibration steps, enabling single-molecule-level analysis of the enzymatic reaction on the membrane.

Our results reveal that the 2D membrane environment can facilitate biological reactions. Specifically, the turnover rate of membrane-bound TEV protease is largely enhanced in the early phase of the reaction compared to both the solution-phase reaction and the reaction in which the substrate is membrane-bound but the enzyme remains in solution. However, this elevated turnover rate declines rapidly as the protease depletes nearby substrates, indicating that the system enters a diffusion-limited regime soon after membrane recruitment.

To further dissect this behavior, we modulated the membrane affinity of TEV protease by varying the length of its His tag and adjusting imidazole concentrations. This created a continuum of enzyme–membrane interaction modes, ranging from strong, permanent membrane anchoring to transient surface-hopping and purely solution-based collisions. Among these modes, the surface-hopping mechanism produced the highest overall enzymatic reaction rate, demonstrating that moderate and dynamic membrane association can help overcome diffusion limitations. These findings emphasize the importance of membrane affinity in tuning enzymatic efficiency at the membrane interface. To optimally exploit the membrane environment, enzymes should possess moderate affinity that allows repeated recruitment and rebinding, enabling them to locally deplete substrate multiple times.

Our results further suggest a broader principle underlying membrane-associated signaling. Adaptor proteins such as Grb2, which link receptor tyrosine kinases (RTKs) to guanine nucleotide exchange factors like SOS, provide an effective mechanism for dynamic membrane recruitment. Grb2 uses both SH2 and SH3 domains to bind phosphotyrosines and proline-rich domains, respectively. These moderate-affinity interactions (with dissociation constants in the micromolar range) contribute additively to the overall binding free energy and enable extended membrane dwell times through serial interactions.^56^ The synergy of SH2- and SH3-mediated interactions, combined with a surface-hopping mechanism, may underlie the rapid and efficient activation of membrane-associated G proteins in vivo. This dynamic recruitment strategy may imply a broader principle for overcoming diffusion limitations and enabling efficient signal transduction at membrane interfaces.

To investigate membrane-mediated reaction kinetics, we reconstituted the TEV proteolytic cleavage reaction on supported lipid bilayers (SLBs), as shown in Figure 1B. The substrate was designed with an N-terminal His_6_ tag, a TEV cleavage site, and a C-terminal GFP, which enabled fluorescence-based monitoring of the reaction. Cleavage of the TEV recognition site releases GFP into the bulk solution. Because TIRF microscopy selectively detects fluorescence near the membrane, substrate cleavage was observed as a time-dependent decrease in GFP fluorescence on the SLB. This loss in signal was used to quantify reaction kinetics.

**Figure 1:**
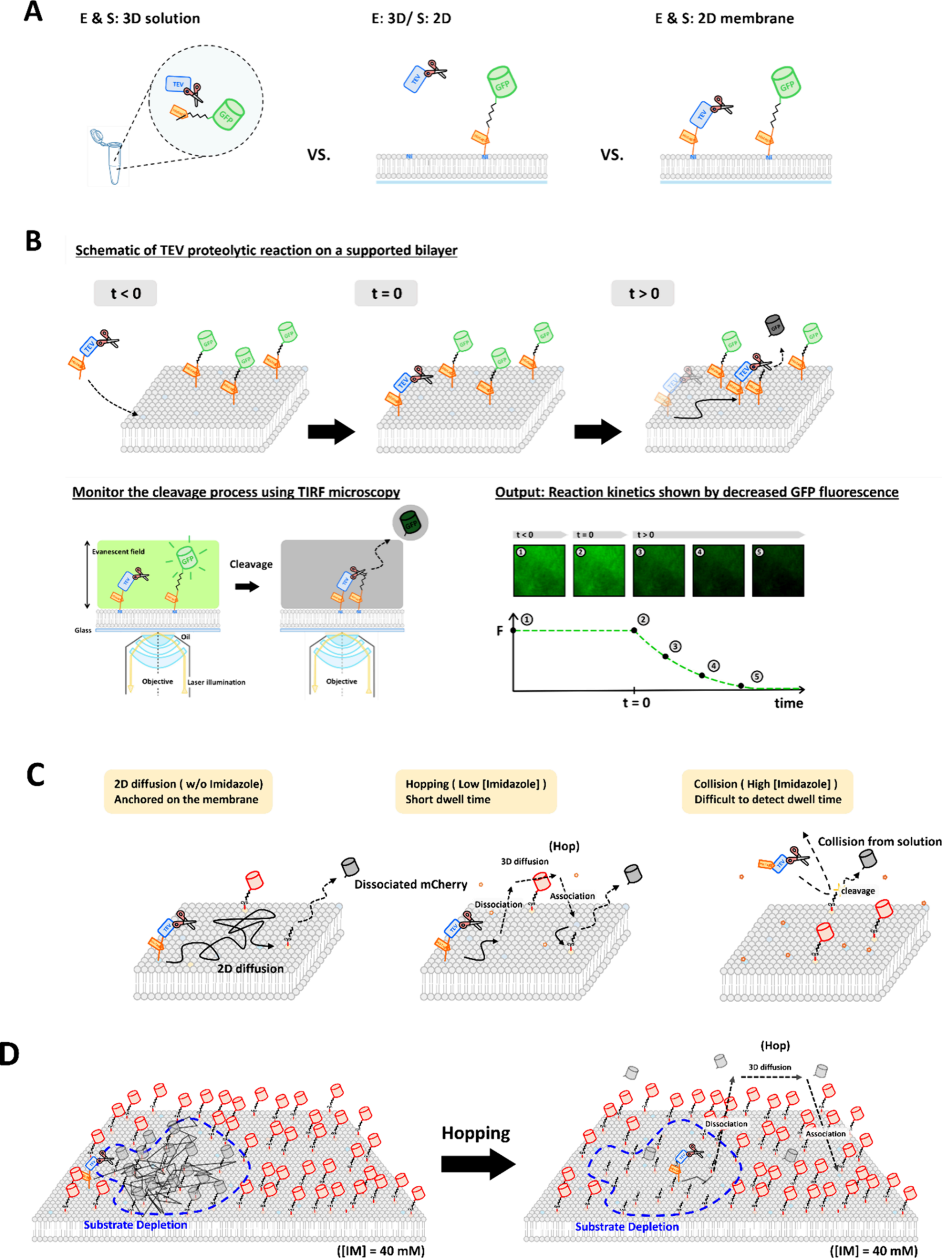
(A) Comparison of TEV proteolytic reactions in different scenarios. (B) Schematic of the TEV proteolytic reaction reconstituted on the SLB. His-tagged GFP is anchored to the SLB containing 8% Ni-NTA DOGS. A TEV cleavage site is positioned between the His-tag and GFP. His-tagged TEV is recruited to the SLB via His-tag chemistry and undergoes lateral diffusion. TEV recognizes and cleaves the substrate at the cleavage site, releasing GFP from the SLB. The enzyme turnover rate (corresponding to real-time reactivity) is determined by monitoring the decrease in GFP fluorescence on the membrane surface. (C) Modulation of His_4_-TEV affinity to the membrane by adjusting the interaction between the His-tag and Ni-NTA lipids. (D) Model of the hopping mechanism. After recruitment to the membrane, TEV undergoes 2D diffusion and reacts with nearby substrates. As local substrate depletion occurs, further cleavage is limited by the slow diffusion of substrates on the SLB. Imidazole can displace His_4_-TEV from Ni-NTA lipids, releasing TEV into solution, where it undergoes faster 3D diffusion and is recruited back to regions with higher substrate density. Repeated cycles of this hopping mechanism enable TEV to overcome local substrate depletion and extend the range of the proteolytic reaction.

To convert GFP fluorescence intensity into substrate surface density, we performed calibration using single-molecule imaging with an EMCCD camera (Figure S1). A linear correlation between fluorescence intensity and molecular count was established. Additionally, we used the ratio of total intensities across different EMCCD settings, measured from images with substrate densities at or above the single-molecule level, to enable accurate quantification of the number of GFP-tagged substrates per square micrometer.

The substrates were anchored to the SLB through His-tag/Ni-NTA interactions using 4–8 mol % Ni-NTA lipids. FRAP measurements confirmed that the substrates undergo two-dimensional Brownian motion on the membrane (Figure S2). TEV protease, labeled with Alexa Fluor 647 via NHS ester chemistry, was recruited to the SLB through its His-tag. Its fluorescence, detected by TIRF microscopy, was calibrated in the same manner as the GFP-tagged substrate to determine TEV surface density (Figures S1 and S8).

The proteolytic cleavage reaction begins immediately after TEV is recruited to the SLB. As shown in Figure 2A, the number of TEV molecules on the SLB increases rapidly at the start and reaches a plateau within approximately 10 min, mimicking signal transduction dynamics following receptor activation. Because TEV lacks autoinhibition, the onset of recruitment is defined as time zero for the proteolytic cleavage kinetics.

**Figure 2:**
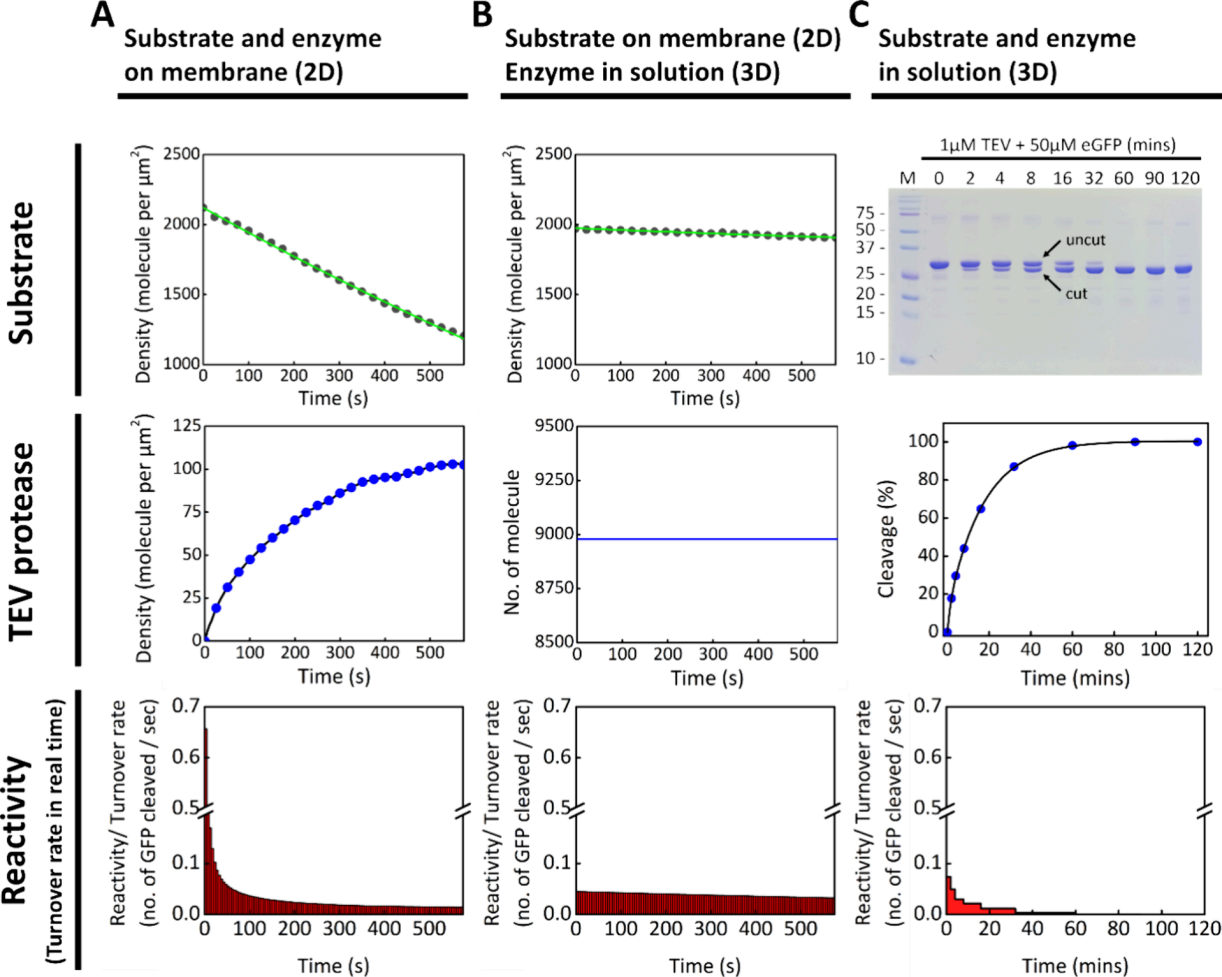
Enzymatic reaction facilitated initially by the 2D surface through higher local concentration. (A) TEV proteolytic cleavage reaction on the SLB. Fluorescently labeled His_7_-TEV is recruited to the SLB (blue dots). GFP anchored on the membrane is released from the surface due to the proteolytic cleavage reaction (black dots). (B) TEV proteolytic cleavage reaction between 2D and 3D systems. Cleavage occurs when TEV in solution collides with the substrate anchored on the SLB. The number of non-His-tagged TEV molecules distributed within a 100 nm-thick volume above the SLB is calculated to be ∼9000 in the imaging area, based on the concentration in the bulk solution. (C) TEV proteolytic cleavage reaction in solution. TEV (final concentration: 1 μM) is mixed with GFP (final concentration: 50 μM) in 0.1 M PB buffer. SDS-PAGE is used to track the kinetics of the cleavage reaction. Enzyme turnover rate is determined by calculating the ratio of cleaved to uncleaved substrate band intensities using ImageJ. A visual break in the *y*-axis is used between 0.2 and 0.5 GFP molecules cleaved per second to accommodate the data range.

Once bound, TEV diffuses laterally on the 2D membrane surface, encounters nearby GFP-tagged substrates, and cleaves the TEV recognition site. The release of GFP from the SLB leads to a decrease in GFP fluorescence, which is monitored as a readout of substrate cleavage. A control experiment without TEV confirmed that fluorescence loss is specific to proteolytic activity, as it shows negligible decay (Figure S3A).

TIRF fluorescence images of both GFP and TEV are acquired every 5 s to track the reaction in real time. Fluorescence intensities are converted into molecular counts, allowing determination of the number of GFP substrates at each time point. The difference in substrate number between successive time points reflects the number of cleaved GFP molecules. By dividing this value by the instantaneous TEV count on the SLB, we calculate the real-time enzymatic reactivity of TEV throughout the reaction.

In Figure 3A and 3B, the surface densities of GFP substrate on the SLB were prepared at approximately 2000 and 1000 molecules/μm^2^, respectively. To generate different TEV densities on the membrane (100, 50, and 20 molecules/μm^2^), the GFP-anchored SLBs were incubated with TEV solutions at varying concentrations. Samples with higher TEV recruitment exhibited a more rapid decline in GFP substrate density, indicating faster cleavage kinetics. When the ratio of GFP substrate to TEV molecules per μm^2^ is high, the initial turnover rate per TEV molecule also increases. Specifically, TEV exhibited turnover rates of 0.66, 3.08, and 4.66 molecules per second when the substrate-to-enzyme ratios were 20, 40, and 100, respectively. These values suggest that, under these conditions, the GFP substrate is in excess, allowing TEV to process more substrate molecules without immediate limitation. The real-time turnover rate of TEV in each condition declines rapidly after its recruitment to the SLB. This observation implies that local depletion of GFP substrates occurs shortly after the reaction begins, and that the proteolytic cleavage becomes diffusion-limited on the SLB surface.

**Figure 3:**
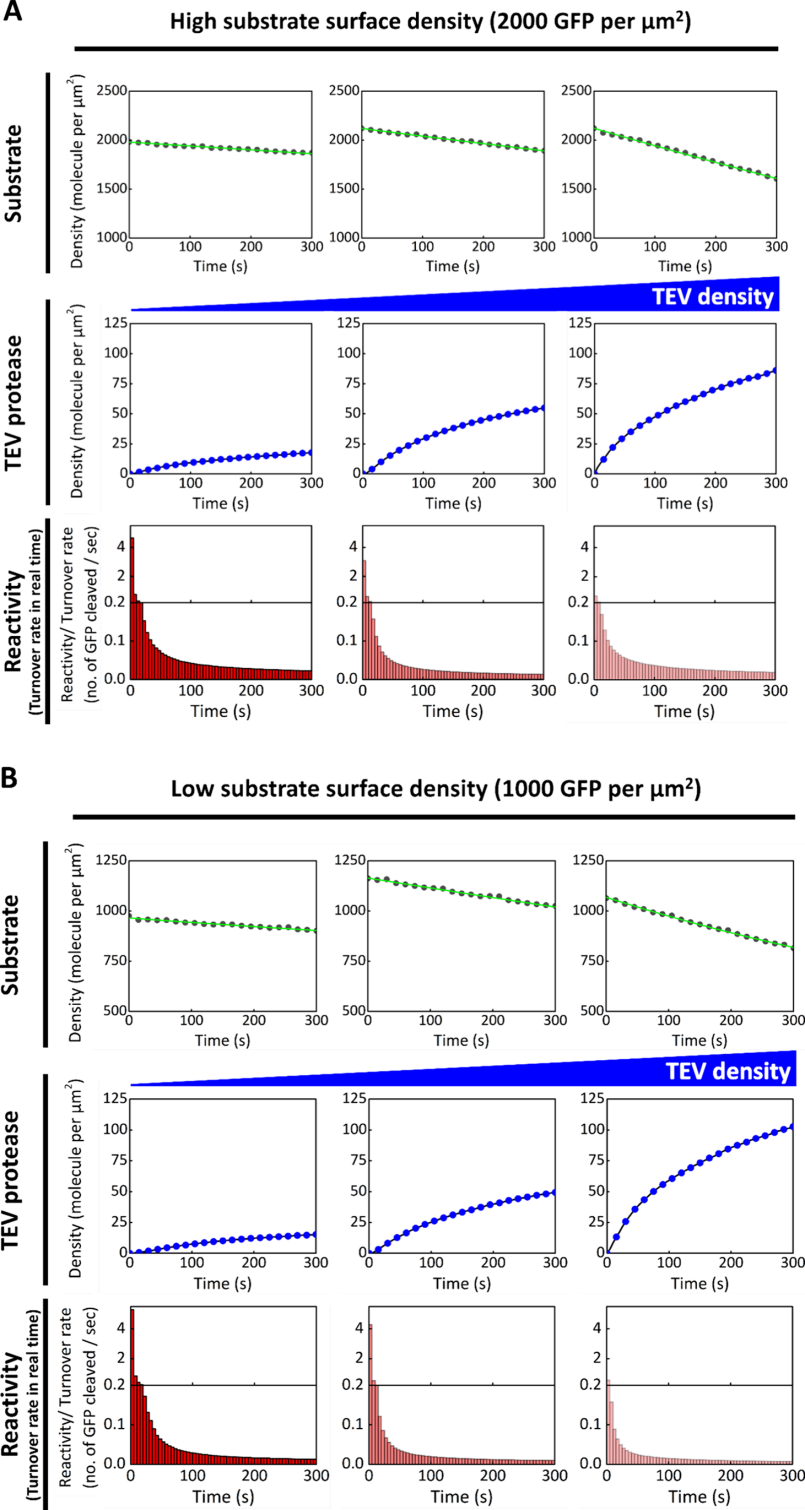
Turnover rate of TEV in real time depends on its surface density. During the reaction, the number of GFP molecules on the SLB decreases due to proteolytic cleavage by TEV (black dot). After the injection of His_7_-TEV into the sample chamber, His_7_-TEV is recruited to the membrane via His-tag chemistry (blue dot). The time course of TEV turnover rate is shown by the red bar diagram. Each bar indicates the average number of substrate molecules cleaved per second by one TEV protease. The real-time turnover rate depends on the surface density of the enzyme and reflects competition for substrates among enzymes. The data demonstrate that as more TEV proteases are recruited to the membrane, the real-time turnover rate per enzyme decreases due to the limited number of substrates available to each enzyme. The surface densities of the substrate are controlled at approximately 2000 and 1000 molecules per μm^2^ in (A) and (B), respectively, and both show similar trends in real-time activity across three different final TEV densities. Statistical analyses of the experiments are provided in Figure S10 and S11.

To explore the upper bound of the TEV turnover rate on the SLB without competition for a limited number of GFP substrates, we constructed a proteolytic reaction with TEV at the single-molecule level (Figure 4). The number of TEV molecules recruited to the SLB was controlled between 400 and 500 within the observed area (55 μm × 55 μm) by incubating 0.05 nM TEV solution with the GFP-anchored SLB. Compared to the bulk experiments in Figure 3, the dependence of the initial TEV turnover rate on the GFP-to-TEV ratio leads to higher initial turnover rates in the single-molecule experiments. In this setup, the GFP-to-TEV ratio is significantly higher than in bulk conditions, resulting in an initial turnover rate exceeding ∼80 molecules per second. Similar to the bulk observations, the TEV turnover rate declines rapidly within a few seconds, suggesting that the reaction quickly reaches a diffusion-limited regime on the SLB.

**Figure 4:**
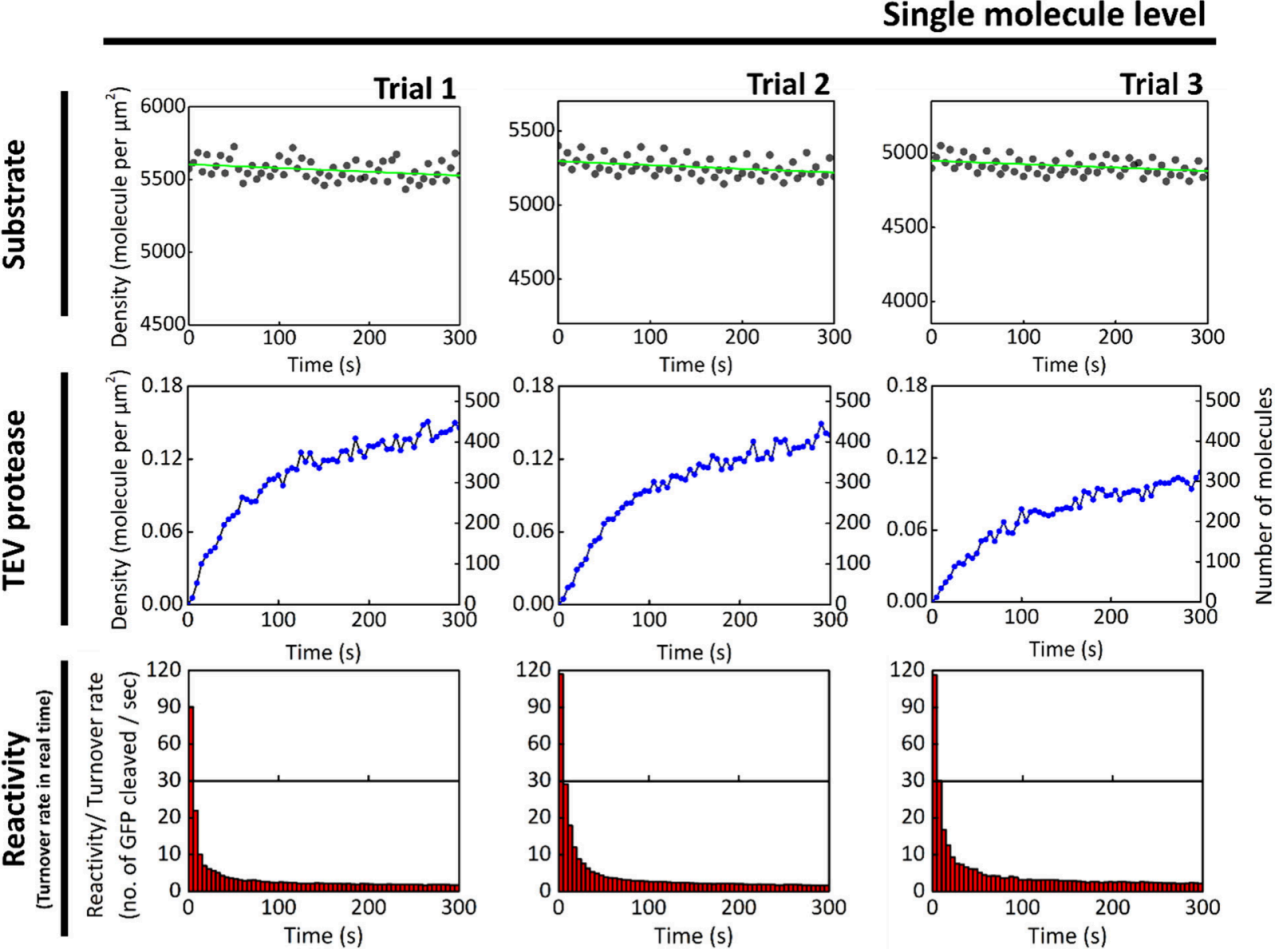
Initial turnover rate of TEV is markedly enhanced by the 2D environment of the SLB when TEV is at the single-molecule level and substrates are in large excess at the start of the reaction. The initial turnover rate increases significantly when both TEV and its substrates are anchored to the SLB and substrate competition among enzymes is minimal during the initial phase of the reaction. The surface density of GFP-tagged substrates on the SLB is controlled at 5000 molecules per μm^2^ (black dot), while the number of TEV molecules recruited to the membrane is controlled at 400–500 (a surface density of approximately 0.12 molecules per μm^2^) within the imaging field (blue dot). The initial turnover rate of TEV exceeds 100 substrate molecules per second (red bar). Statistical analyses of the experiments are provided in Figure S10 and S11.

Given that the dissociation of fluorescent protein from the membrane serves as the readout for reaction kinetics at the interface, potential drawbacks associated with fluorescent proteins must be carefully considered. These include: (1) photobleaching, which can artificially reduce signal intensity over time; (2) nonspecific membrane binding, which may bias kinetic interpretations; and (3) intrinsic photophysical or photochemical properties specific to each fluorescent protein, such as oligomerization, transitions to nonemissive (dark) states, or conversion to alternative fluorescent states. The first two concerns were addressed through control experiments shown below (Figures S3, S4, S5 and S8), while the third was evaluated by comparing different fluorescent proteins (Figure S9). Overall, the major limitations of fluorescent protein-based readouts have been carefully assessed in this study. Looking ahead, self-labeling protein tags may offer a promising alternative for further improving signal fidelity and system performance.

Because both TEV and the GFP substrate are anchored to the SLB via His-tag chemistry, there is a potential concern that they may compete for Ni-NTA binding sites. Such competition could lead to dissociation of the GFP substrate from the SLB, resulting in an overestimation of TEV turnover rate. To verify that the observed decrease in GFP fluorescence is due to proteolytic cleavage rather than substrate displacement, we used a catalytically inactive mutant of TEV (TEV^C151A^), in which cysteine at position 151—part of the catalytic triad—was mutated to alanine. This mutation has been reported to abolish enzymatic activity without disrupting the overall protein structure.^57^ SDS-PAGE analysis confirmed the inactivity of TEV^C151A^ (Figure S5): the protein migrated at ∼71 kDa, indicating that the maltose-binding protein (MBP, 43 kDa) fusion tag remained uncleaved. In contrast, the functional TEV undergoes autocleavage at the TEV recognition site between MBP and TEV. The presence of uncleaved MBP thus confirms that TEV^C151A^ is catalytically inactive.

However, the MBP tag also masks the His_7_ tag, preventing TEV^C151A^ from binding to the SLB via Ni-NTA. To resolve this, we removed the MBP sequence and used a His_7_–TEV^C151A^ fusion protein as a control. When introduced under the same experimental conditions, His_7_–TEV^C151A^ was successfully recruited to the SLB bearing GFP substrate. In this control experiment, GFP fluorescence remained nearly constant during TEV^C151A^ recruitment (Figure S4), confirming that the fluorescence decrease observed with functional TEV results from proteolytic cleavage rather than competitive displacement from the SLB.

In the solution-phase (3D) experiments, TEV and GFP substrate were prepared at micromolar concentrations (1 μM and 50 μM, respectively) in PB buffer (pH 8), with a TEV-to-GFP molar ratio of 1:50. These concentrations are approximately 2 orders of magnitude higher than those used in the 2D SLB experiments. The kinetics of the cleavage reaction were monitored by collecting 2 μL aliquots at different time points and flash-freezing them for SDS-PAGE analysis. As shown in Figure 2C, the gel reveals that most GFP substrates were cleaved within 90 min, as indicated by the appearance of a band corresponding to the cleaved, lower-molecular-weight product. The percentage of cleaved substrate was quantified using ImageJ and used to plot the reaction kinetics.

Based on these data, the average real-time turnover rate of TEV in solution was calculated. In the first 120 s, each TEV molecule cleaved an average of only 0.074 GFP substrates. In contrast, the corresponding turnover rate in the 2D SLB system during the same time window was about 0.093 molecules per second—despite using enzyme and substrate concentrations that were 1–2 orders of magnitude lower than those in the 3D solution. This suggests that membrane confinement and localization may enhance enzymatic efficiency, even in the presence of substrate depletion.

To further investigate the role of the membrane interface, we separated TEV and the GFP substrate into different dimensionalities. The membrane affinity of TEV was eliminated by removing its His_7_ tag. This was accomplished by inserting a Factor Xa recognition site between TEV and the His_7_ tag and cleaving the tag during purification. The resulting non-His-tagged TEV was introduced into the bulk solution above the SLB, where GFP substrate remained membrane-anchored via His-tag chemistry. In this configuration, the reaction depends on three-dimensional diffusion and collision between TEV and the SLB-bound substrate. The number of TEV molecules distributed within 100 nm of the SLB surface was estimated based on the bulk concentration. For a protein the size of BSA (∼66 kDa) with a diffusion coefficient of 60 μm^2^/s, the root-mean-square displacement over a 50 ms exposure is ∼4.2 μm, justifying 100 nm as a conservative estimate for calculating the effective 2D density. Under these conditions, GFP fluorescence showed only a slight decrease over time, and the TEV turnover rate remained nearly constant at ∼0.045 molecules per second (Figure 2B).

In the 2D experiment, we observed a rapid decrease in TEV turnover rate within 5 s of initiating the reaction, consistent with a diffusion-limited process on the membrane. In biological systems, signaling cascades often rely on low-affinity (*K*_D_ ≈ 1–100 μM), reversible interactions between cytoplasmic adaptor proteins and membrane receptors. After recruitment, adaptor proteins do not remain confined to the plasma membrane but instead move between receptors via a protein hopping mechanism, enabling sequential binding events. To mimic this dynamic behavior, imidazole was introduced to modulate the affinity between the His-tag and Ni-NTA lipids, thereby controlling the mode of molecular motion on the SLB.

Four SLB conditions were prepared by adjusting the imidazole concentration in TBS buffer to 0 mM, 10 mM, 20 mM, and 40 mM. A 0.05 nM His_4_-TEV solution was injected into each condition, allowing us to monitor the membrane association of TEV using total internal reflection fluorescence (TIRF) microscopy. By tracking the recruitment and disappearance of individual His_4_-TEV molecules—driven by imidazole competition and exit from the TIRF illumination field—we obtained dwell time distributions under each condition. As shown in Figure 5, higher imidazole concentrations resulted in shorter dwell times, consistent with weaker His-tag/Ni-NTA interactions.

**Figure 5:**
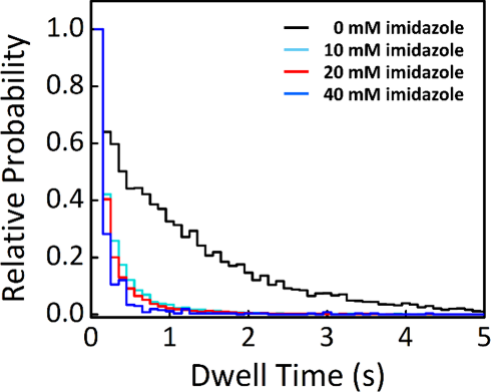
Modulation of His_4_-TEV dwell time on the membrane by varying the concentration of imidazole. The distribution of the dwell time for His_4_-TEV on the membrane is measured at four different concentrations of imidazole, shown in black (0 mM), cyan (10 mM), red (20 mM), and blue (40 mM). Imidazole competes with the His-tag for binding to the Ni-NTA lipid, causing the dissociation of His_4_-TEV into the solution above and shortening its dwell time on the membrane. The histogram shows that the dwell time of His_4_-TEV greater than 0.5 s is highly populated when there is no imidazole in the system, while the distribution of dwell times shifts to the short time scale (<0.5 s) as the concentration of imidazole increases.

After confirming that imidazole can modulate membrane affinity to enable the hopping mechanism, we applied it to the reconstituted proteolytic cleavage reaction on the SLB to investigate how this mode of motion affects reaction rates. However, because the GFP substrate is also anchored to the SLB via His-tag chemistry, the introduction of imidazole would destabilize its binding. To avoid this complication, we redesigned the substrate: a cysteine residue was added to its N-terminus, and GFP was replaced with mCherry as the fluorescent probe. The mCherry substrate was anchored to the SLB by covalently coupling the engineered cysteine to maleimide-functionalized lipids (PE MCC), as illustrated in Figure 1C. Since mCherry has no native cysteine residues, the engineered cysteine provides the sole anchoring point between the substrate and the SLB.

Using this system, we mimicked three distinct scenarios for His_4_-TEV interaction with the SLB: 2D diffusion, hop diffusion, and solution-phase collision, corresponding to imidazole concentrations of 0 mM, 40 mM, and 200 mM, respectively (Figure 1C). At 0 mM imidazole, the His-tag binds strongly to the Ni-NTA lipids, allowing stable recruitment of His_4_-TEV and lateral diffusion on the membrane. Under these conditions, the TEV turnover rate initially peaks at ∼0.78 molecules per second but rapidly declines due to diffusion-limited substrate depletion. When 40 mM imidazole is introduced, the membrane affinity is weakened, resulting in hop diffusion: TEV transiently associates with the SLB, diffuses laterally, then dissociates and rebinds. This cycling extends the effective range of each TEV molecule and sustains a relatively high turnover rate. The TEV surface density reaches a plateau, indicating a dynamic equilibrium between recruitment and dissociation. As shown in the left portion of Figure 6B, this hopping mode delays substrate depletion and maintains elevated enzymatic activity compared to the 0 mM imidazole condition. At 200 mM imidazole, His_4_-TEV is unable to bind the SLB due to strong competition from imidazole in solution. In this case, the proteolytic reaction occurs solely through transient collisions between TEV and the membrane-anchored substrate, leading to a substantially reduced turnover rate (Figure 6).

**Figure 6:**
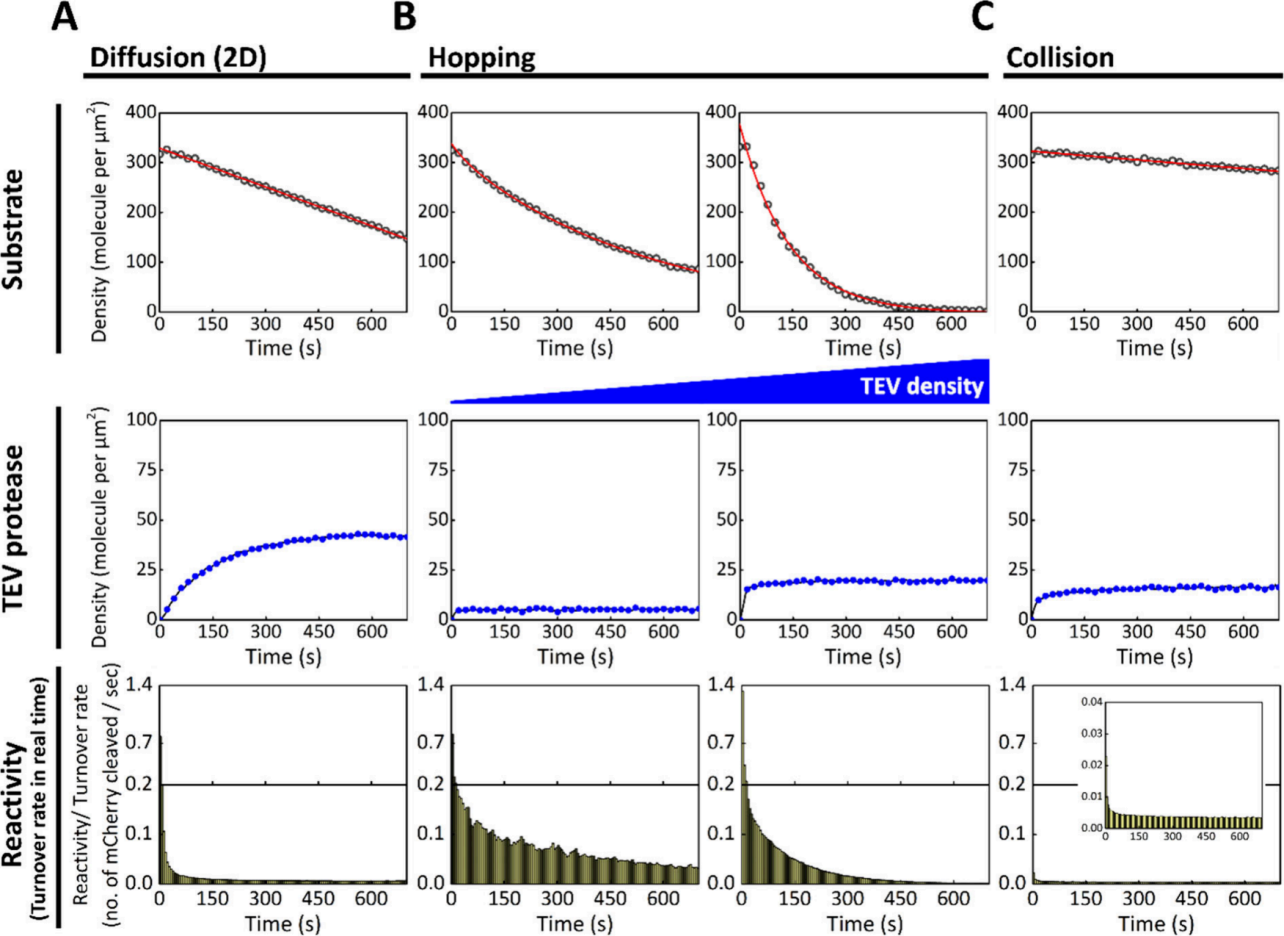
Hopping mechanism allows the enzyme to escape from localized movement due to short dwell times on the membrane, unlike molecules that remain permanently anchored. mCherry is anchored to the membrane via maleimide chemistry, with a TEV cutting site between the introduced cysteine at the N-terminus and mCherry. The surface density of mCherry is controlled at about 350 molecules per μm^2^. The type of movement on the membrane is changed from 2D diffusion to the hopping model by modulating the affinity between the His-tag and Ni-NTA lipid through the addition of imidazole. TEV will eventually collide with the membrane from solution when the imidazole concentration is high enough. (A) Without imidazole ([imidazole] = 0 mM) in the system, the interaction between the His-tag and Ni-NTA lipid is strong. His_4_-TEV is recruited to the membrane via His-tag chemistry and undergoes 2D diffusion on the membrane. The initial TEV turnover rate is enhanced by the 2D surface (to about 0.7 substrate molecules cleaved per second per TEV), but it soon drops rapidly due to diffusion-limited conditions between enzymes and substrates on the membrane. (B) A low concentration of imidazole ([imidazole] = 40 mM) reduces the affinity between His_4_-TEV and the SLB containing 4% Ni-NTA lipid. This moderate affinity results in the hopping movement of TEV on the SLB. Under this condition, TEV undergoes cycles of recruitment to the SLB, 2D diffusion, and dissociation from the SLB, effectively expanding the range of the proteolytic cleavage reaction by a single TEV molecule. The leveling off of TEV density reflects equilibrium between its dissociation from and recruitment to the membrane. TEV can thus maintain a relatively high turnover rate by avoiding local substrate depletion (left panel). However, in the presence of a higher density of TEV, the turnover rate drops quickly due to rapid substrate depletion. The same experiments using a different fluorescent protein, mScarlet, are shown in Figure S9. (C) His_4_-TEV is no longer recruited to the membrane at high imidazole concentrations ([imidazole] = 200 mM). In this case, TEV reacts with the substrate only through collisions from solution, resulting in a relatively low turnover rate. A surface density of TEV comparable to the hopping condition in (B) is achieved by adjusting the concentration of protein in the sample injection. Statistical analyses of the experiments are provided in Figures S10.

*Membrane Localization Enhances Reaction Efficiency*. We reconstituted a model TEV proteolytic cleavage reaction on the SLB to directly study biochemical kinetics at the membrane interface, without additional modulation of enzymatic turnover via allostery or cofactors. In the first part of the study, both the enzyme (TEV) and the substrate (an eGFP-tagged TEV cleavage sequence) were prepared either on the SLB or in the bulk solution, resulting in three distinct scenarios (Figure 1A). For the membrane-associated reaction, TEV cleavage leads to the dissociation of the eGFP fusion tag into the bulk solution. The corresponding decrease in fluorescence intensity on the SLB is monitored in real time by TIRF microscopy and used to track the reaction kinetics (Figure 1B).

Among the three configurations, the two cases in which both TEV and the substrate are located either on the SLB or entirely in solution produce clear cleavage kinetics (Figures 2A and 2C), despite large differences in reactant concentrations. In the 2D SLB system, TEV and substrate concentrations are ∼100 nM or lower due to strong His-tag-mediated membrane binding. In contrast, the 3D solution-phase system uses much higher micromolar concentrations. Nevertheless, the average TEV turnover rate on the SLB, measured during the first 2 min of the reaction, is approximately 0.093 substrates per second—comparable to the initial rate observed in solution. These results suggest that strong membrane affinity can compensate for lower reactant concentrations by enhancing local reaction efficiency. In contrast, when the enzyme is in solution and the substrate is anchored to the SLB, the reaction proceeds minimally (Figure 2B). This observation illustrates how membrane recruitment can gate reactivity at the membrane surface: TEV and its substrate, even at nanomolar concentrations in the cytosol, are unlikely to undergo efficient reactions through random collisions alone without colocalization on the membrane.

*Diffusion-Limited Turnover on the Membrane*. The kinetics of the TEV proteolytic cleavage reaction on the SLB were further investigated in detail (Figures 3 and 4). In Figure 3, cleavage reactions were reconstituted on SLBs with two different GFP substrate densities—2000 and 1000 molecules per μm^2^—using comparable concentrations of TEV introduced from solution. In both conditions, increasing the number of TEV molecules led to faster reaction kinetics, and a higher substrate-to-enzyme ratio produced a higher initial enzyme turnover rate. Notably, similar enzymatic reactivities were observed TEV densities across the two substrate conditions (between part A and B of Figure 3), suggesting that GFP substrates are rapidly depleted in the vicinity of the enzyme and that their replenishment is limited by slow diffusion on the SLB.

To explore the upper limit of TEV turnover rate under substrate-excess conditions, we further increased the substrate-to-enzyme ratio by conducting the reaction at the single-molecule level. Biological reactions at the single-molecule level are particularly relevant for membrane-associated signaling, where activation often begins with very small numbers of molecules. For example, T cell receptor (TCR) activation can be triggered by the engagement of just a few ligands, highlighting the biological importance of reactions that operate effectively under such sparse conditions. Recent studies have demonstrated that localized single-molecule interactions at the TCR are sufficient to initiate signaling cascades.^58,59^ As shown in Figure 4, a highly diluted TEV solution—200-fold lower than that used in the experiments in Figure 3—was added to the SLB to achieve sparse, single-molecule enzyme occupancy within the imaging field. Under these conditions, the initial turnover rate of TEV exceeded 100 molecules per second, more than 20-fold higher than the rate observed under low TEV density in Figure 3. Given that the GFP substrate density in this experiment was only 3- to 5-fold higher than those used in Figure 3, while the TEV density was 120-fold lower, the observed ∼20-fold increase in turnover rate is disproportionately small. This deviation from first-order scaling underscores a limitation imposed by the two-dimensional geometry of the membrane, where substrate access to the enzyme’s active site is restricted to the plane of the SLB.

Besides the deviation from first-order dependence on enzyme and substrate densities for initial turnover rates, the TEV turnover rate also declines sharply over time, as shown in the kinetic traces in Figures 3 and 4. In these experiments, both the enzyme and the substrate are confined to the SLB. Their effective concentrations—estimated using a 5 nm interfacial thickness, as described by Leonard et al.^25^—are approximately 28 μM for TEV and 700 μM for the GFP substrate. Based on these concentrations, faster reaction kinetics would be expected compared to the 3D solution-phase conditions used in Figure 2C. However, the observed slower kinetics and rapid decay in turnover rate in Figures 3 and 4 are consistent with diffusion-limited substrate access on the membrane.

This slowdown can be attributed to the significantly reduced diffusion of molecules on the membrane. Membrane-bound proteins typically diffuse 2 orders of magnitude more slowly than their soluble counterparts. For instance, GFP has a diffusion coefficient of ∼30 μm^2^/s in the cytoplasm,^60^ but this drops to ∼0.2 μm^2^/s when localized to the membrane—a 150-fold reduction, as reported by Leonard et al.^25^ and others.^33^ Similarly, Ras diffuses at ∼20 μm^2^/s in the cytoplasm^61^ but slows to ∼1 μm^2^/s when membrane-anchored.^14,62^ Once TEV is recruited to the membrane, it rapidly cleaves nearby GFP substrates, but fresh substrate access is restricted by the slow diffusion of membrane components. This diffusion-limited encounter frequency explains the rapid drop in enzyme activity observed in Figures 3 and 4.

*Overcoming Diffusion Constraints via Membrane Hopping*. This membrane-imposed kinetic limitation resembles regulatory strategies used in biological signaling. Many peripheral membrane proteins exhibit moderate affinity for the membrane and bind transiently upon activation. For example, the SOS protein in the MAPK signaling pathway can efficiently activate membrane-anchored Ras without being permanently attached to the membrane.^63–65^ To experimentally test the effect of reduced membrane residence time, we modulated TEV’s membrane affinity using imidazole, which weakens His-tag interactions with Ni-NTA lipids. TIRF-based single-molecule tracking was used to measure the 2D Brownian motion and membrane dwell times of fluorescently labeled TEV. As shown in Figure 5, increasing imidazole concentrations resulted in shorter dwell times. The corresponding reduction in TEV track length is presented in Figure S7. These observations confirmed that TEV exhibits a hopping-like behavior on the membrane under moderate-affinity conditions.

Because imidazole disrupts substrate anchoring via His-tag chemistry, we employed an orthogonal anchoring strategy for the TEV substrate. The substrate was redesigned by replacing GFP with mCherry and introducing a cysteine residue at the C-terminus, just upstream of the TEV cleavage site. This engineered cysteine allowed covalent attachment of the substrate to the SLB through maleimide-functionalized lipids. Upon cleavage by TEV, the mCherry fragment is released into the bulk solution, analogous to the GFP-based system. To assess the role of membrane hopping, three experimental conditions were prepared (Figure 1C): (1) 0 mM imidazole, where both TEV and the substrate are anchored to the SLB; (2) 40 mM imidazole to reduce TEV’s membrane affinity and induce hopping; and (3) 200 mM imidazole to prevent TEV membrane recruitment entirely, limiting interactions to transient solution-phase collisions. Molecular densities in these experiments were quantified using the same approach as in previous sections but calibrated separately for mCherry due to its distinct fluorescence properties.

Surprisingly, under hopping conditions (40 mM imidazole), TEV displayed faster reaction kinetics than in the condition where both enzyme and substrate were membrane-bound. At higher TEV densities, nearly all mCherry substrates were cleaved within 10 min (Figure 6). In contrast, reactions relying on TEV–membrane collisions at 200 mM imidazole exhibited much slower kinetics. Notably, TEV with hopping capability maintained higher turnover rates and effectively overcame the membrane diffusion limit (Figure 1D and 6B). Although initial activities were similar in the hopping and membrane-anchored scenarios, turnover rates for membrane-anchored TEV dropped rapidly due to diffusion constraints. The high density of hopping TEV also led to substrate competition, reflected in steep declines in both mCherry substrate density and enzymatic turnover rate. Meanwhile, the solution-collision condition failed to sustain efficient cleavage, indicating that mere collision frequency is insufficient to drive robust membrane-associated reactions. These results suggest that membrane affinity acts as a molecular switch to initiate or regulate reactions at the membrane interface in biological systems. The relative turnover rates from the different models are summarized in the table of content.

*Biological Relevance of Transient Membrane Interactions*. In this study, we observed that molecules exhibiting hopping behavior on the membrane can overcome the diffusion limitation associated with permanent membrane anchoring. Many biomolecules involved in signal transduction exhibit moderate membrane affinity, often characterized by micromolar dissociation constants. Examples include the interaction between the SH2 (Src Homology 2) domain of Grb2 and phosphotyrosines, as well as the binding between GPCRs and G-proteins.^66,67^ Molecules that hop on the membrane have this intermediate level of affinity, allowing transient association and increased spatial exploration. Previous studies have shown that adaptor proteins can reversibly bind to receptors anchored on the membrane, supporting this model of dynamic membrane engagement.^68,69^ For example, Grb2 is recruited to the membrane through interactions with phosphotyrosines on the cytoplasmic tail of EGFR. Due to the micromolar dissociation constant between Grb2 and EGFR, Grb2 can dynamically hop among phosphotyrosines across multiple receptors. This transient binding behavior increases the likelihood of encounters with downstream signaling proteins and enhances signal propagation efficiency.^56,70^

In summary, we *in vitro* reconstituted the proteolytic cleavage reaction on the SLB as the model system to interrogate the role of the membrane mediating the biological reaction at the interface. The dissociation of the fluorescence tag after the proteolytic cleavage reaction allows us to real-time monitor the reaction on the membrane at the single-molecule level. Our work systematically compares the kinetics from different scenarios of the enzyme and the substrate prepared at the interface of the membrane and the bulk solution. From the enzymatic turnover rate of both the enzyme and the substrate anchored on the membrane, it shows that the membrane can increase the interaction between the enzyme and the substrate and give the relatively high enzymatic turnover rate at first. However, the reaction on the membrane is soon subjected to the diffusion limit from the membrane and results in the fast drop of the enzymatic reactivity. To address this limitation, we further chemically modulate the membrane affinity of the enzyme to generate the enzyme hopping on the membrane. The reaction kinetics from the hopping enzyme shows the fastest kinetics under the similar condition and can overcome the diffusion limit resulting in the extended decay of the enzymatic turnover rate. Our results provide the key evidence to support the idea that the moderate membrane affinity adopted by nature can fully extract the benefits from the membrane for the high efficiency of the biological reactions at the interface. Our study highlights the dual role of membranes in enhancing enzymatic efficiency while imposing diffusion limitations and introduces enzyme hopping as a natural strategy to overcome these constraints. By offering insights into the molecular mechanisms behind membrane-mediated processes, our work lays the foundation for future research on biological reactions at membrane interfaces and their evolutionary adaptations.

## Supplementary Material



## Notes

Detailed descriptions of materials and methods, along with supplementary figures for the calibration procedures, control experiments, and statistical analyses (Figures S1–S11) (PDF)
