# Supplementary material for: Enzymatic Reactions Dictated by the 2D Membrane Environment
Source: J Phys Chem Lett. 2025 Jun 24;16(26):6745–56. doi: 10.1021/acs.jpclett.5c00988 (PMC12235624; doi:10.1021/acs.jpclett.5c00988)
Supplement: Supplementary file 1 [file jz5c00988_si_001.pdf]

Supporting Information

Enzymatic Reactions Dictated by the 2D Membrane Environment

Ru-Hsuan Bai, Chun-Chen Lin, Chun-Wei Lin *

Department of Chemistry, National Tsing Hua University, Hsinchu, Taiwan 300044.

Email: chunweilin@mx.nthu.edu.tw

Contents

Materials and Methods .... page 2-5

Figures S1–S11 .... page 6-17

Materials and Methods

Chemicals

1,2-dioleoyl-sn-glycero-3-phosphocholine (DOPC), 1,2- dioleoyl-sn-glycero-3-[(N-(5-amino-1-carboxypentyl) iminodiacetic acid) succinyl] (Ni^{2+}-NTA-DOGS; nickel salt) and 1,2-dioleoyl-sn-glycero-3-phosphoethanolamine-N-[4-(p-maleimidomethyl) cyclohexane-carboxamide] (PE MCC; sodium salt) were purchased from Avanti Polar Lipids. Alexa Fluor 647 NHS ester was purchased from Lumiprobe. Bovine serum albumin (BSA) was purchased from Sigma-Aldrich. Sulfuric acid (H_2SO_4) and hydrogen peroxide (H_2O_2) were purchased from Honeywell Fluka. Tris-buffered saline (TBS) was purchased from Protech Technology.

Protein purification

GFP-tagged substrate The green fluorescent protein (GFP) sequence with an N-terminal His_6 tag and a TEV protease cleavage site was cloned into the p11X plasmid. The plasmid was transformed into *Escherichia coli* BL21 (DE3) pLysS using the heat shock method. Transformed colonies were inoculated into 1 L of Terrific Broth medium and incubated at 37 °C. Once the culture reached an optical density at 600 nm (OD_{600}) of approximately 0.6, expression was induced with 1 mM isopropyl β-D-1-thiogalactopyranoside (IPTG) and the culture was incubated at 37 °C for 2 h. Cells were harvested by centrifugation at 6000 × g for 20 min, and the supernatant was discarded. The resulting pellet was resuspended in 50 mL Ni-NTA buffer (20 mM Tris–HCl, 500 mM NaCl, 20 mM imidazole, 10% glycerol, pH 8.0) by vortexing. The resuspended cells were lysed by sonication. Cell debris was removed by ultracentrifugation at 15,000 × g for 30 min. The clarified lysate was loaded onto a HisTrap FF column (GE Healthcare) pre-equilibrated with Ni-NTA buffer. After washing to remove non-specifically bound proteins, GFP was eluted with Ni-elution buffer (20 mM Tris–HCl, 500 mM NaCl, 500 mM imidazole, 10% glycerol, pH 8.0). The eluate was concentrated to 500 µL and buffer-exchanged to size exclusion buffer (0.1 M phosphate buffer, pH 8.0) using an Amicon Ultra Centrifugal Filter Unit (10 kDa molecular weight cutoff [MWCO]; Millipore). The sample was then loaded onto a Superdex 75 10/300 GL column (GE Healthcare) equilibrated with the same buffer. Fractions containing GFP were collected, and protein purity was confirmed by SDS-PAGE. The final concentration was determined, and the purified GFP was aliquoted, flash-frozen in liquid nitrogen, and stored at −80 °C.

mCherry-tagged substrate A pET28 vector containing the sequence for His_6–Factor Xa cut site–Cys–TEV cut site–mCherry was transformed into *Escherichia coli* BL21 (DE3) cells. Protein expression and purification using a HisTrap FF column (GE Healthcare) and a Superdex 75 10/300 GL column (GE Healthcare) were performed as described in the GFP-tagged substrate purification section. The N-terminal His_6 tag was removed by treatment with Factor Xa protease, and the cleaved sample was reapplied to the HisTrap FF column. The flow-through, containing the cleaved protein, was collected, aliquoted, flash-frozen, and stored at −80 °C.

Functional TEV pretease The pRK508 plasmid containing the TEV S219V mutant fused to an N-terminal His_7 tag and an MBP fusion tag, with a tobacco etch virus (TEV) cleavage site, was transformed into *Escherichia coli* BL21 (DE3). Successfully transformed colonies were transferred to 1 L of Terrific Broth medium and incubated at 37 °C until the optical density at 600 nm (OD_{600}) reached 0.6. Protein expression was induced with 1 mM IPTG, and the culture was grown at 37 °C for 3 h. The cells were harvested by centrifugation at 6000 × g for 20 min, and the pellet was collected and resuspended in 50 mL buffer A (50 mM HEPES, 1 M NaCl, 20 mM imidazole, 10% glycerol, pH 7.5). The resuspended cells were lysed by sonication, and cell debris was removed by ultracentrifugation at 15,000 × g for 30 min. The clarified supernatant was loaded onto a HisTrap FF column (GE Healthcare) pre-equilibrated with buffer A. After removing non-target proteins, TEV was eluted using buffer C (50 mM HEPES, 50 mM NaCl, 400 mM imidazole, 10% glycerol, pH 7.5). The eluate was concentrated to 5 mL and buffer-exchanged into buffer B (50 mM HEPES, 50 mM NaCl, 20 mM imidazole, 10% glycerol, pH 7.5) using an Amicon Ultra Centrifugal Filter Unit (10 kDa molecular weight cutoff [MWCO]; Millipore).

The concentrated sample was then loaded onto a HiTrap SP FF column (GE Healthcare) to further purify TEV by cation exchange, adjusting the NaCl concentration by altering the ratio of buffer B to buffer D (50 mM HEPES, 400 mM NaCl, 20 mM imidazole, 10% glycerol, pH 7.5). Fractions containing TEV were eluted at approximately 25% buffer D. The eluted protein was subsequently concentrated and buffer-exchanged into size exclusion buffer using an Amicon Ultra Centrifugal Filter Unit (10 kDa MWCO). The sample was then loaded onto a Superdex 75 10/300 GL column (GE Healthcare) equilibrated with size exclusion buffer. Fractions containing purified TEV were collected, and the protein concentration was determined by UV-Vis spectrophotometry. The final protein solution was flash-frozen and stored at −80 °C for subsequent labeling reactions.

Protein labeling

Protein fluorescence labeling was performed at a 1:1 molar ratio (dye:protein). Alexa Fluor 647 NHS ester was dissolved in anhydrous DMSO to prepare a 2 mg/mL dye stock. TEV was concentrated to a volume nine times greater than the dye stock to maintain a 9:1 volume ratio between protein and dye. After mixing, the labeling reaction was carried out at room temperature for 1 hour, followed by incubation at 4 °C for 8 hours. Precipitate was removed from the labeled sample by ultracentrifugation. Subsequently, unreacted dye was removed using an Amicon Ultra Centrifugal Filter (10 kDa molecular weight cutoff [MWCO]; Millipore), and the sample was concentrated. The concentrated protein was further purified by size exclusion chromatography (Superdex 75; GE Healthcare). Protein concentration and labeling efficiency were determined by UV-Vis spectrophotometry. Finally, aliquots of the labeled protein were flash-frozen in liquid nitrogen and stored at −80 °C.

Supported lipid bilayers (SLBs)

Supported lipid bilayers (SLBs) were prepared by vesicle rupture on the glass substrate. Small unilamellar vesicles (SUVs) were composed of a lipid mixture of DOPC and Ni-NTA DOGS at a molar ratio of 92:8 in chloroform. The lipid mixture was evaporated using a rotary evaporator at 40 °C for 3 min to remove chloroform and form a dried lipid film, followed by a nitrogen blow for 15 min to ensure complete drying. 2 mL of deionized water (DI H_2O) were added to the dried lipid film, followed by vortexing and pipetting to resuspend the lipids and form vesicles of heterogeneous sizes. SUVs were generated by sonicating the vesicle suspension with a probe tip sonicator.

The glass substrate was treated with piranha solution (H_2SO_4:H_2O_2 in a 3:1 volume ratio) for 8 min to enhance the hydrophilicity of the surface (glass coverslips, bottom thickness 170 µm ± 5 µm; Ibidi). The cleaned glass substrate was then assembled with a flow chamber (Sticky-Slide VI 0.4; Ibidi). The SUV suspension was mixed with TBS buffer in a 1:1 volume ratio and injected into the assembled chamber, followed by incubation for 25 min to allow SLB formation via vesicle rupture. To block defects in the SLB, 1 mg/mL BSA was incubated in the chamber for 15 min.

GFP was anchored to the Ni-NTA lipids via His-tag–Ni^{2+}-NTA interactions by incubating the protein with the SLB for 20 min. The lateral mobility of GFP on the membrane was examined by fluorescence recovery after photobleaching (FRAP). Protein surface density on the membrane was determined using a calibration curve correlating the average fluorescence intensity in the imaging region with the corresponding protein density (Figure S1 in the Supporting Information).

TIRF Microscopy

Imaging was performed using a Nikon Eclipse Ti inverted microscope equipped with a TIRF system and an iXon electron-multiplying charge-coupled device (EMCCD) camera (Andor Technology). TIRF microscopy was carried out with a Nikon 100× 1.49 NA oil-immersion objective, a TIRF illuminator, a Perfect Focus system, a motorized stage, and a U-N4S four-laser unit (Nikon) as the laser source.

The system was equipped with solid-state lasers operating at 488 nm, 561 nm, and 640 nm, controlled via a built-in acousto-optic tunable filter. Laser powers were set to 5.2 mW (488 nm), 6.9 mW (561 nm), and 7.8 mW (640 nm), measured with the field aperture fully opened. For fluorescence filtering, the 405/488/561/638 nm Quad TIRF filter set (Chroma Technology Corp.) was used. Images were captured using Nikon NIS-Elements software at 5 s intervals with an exposure time of 20 ms.

TEV proteolytic reaction on the SLB

His-tagged GFP with a TEV cleavage site served as both the reaction substrate and the fluorescent signal output. The SLB contained 8 mol% Ni-NTA lipids, a moderately increased proportion used to minimize competition for anchor lipids between the GFP substrate and TEV protease. Upon adding His-tagged TEV to the SLB with the GFP-tagged substrate, TEV was recruited to the membrane and cleaved the recognition site, resulting in the release of GFP from the membrane. The decrease in GFP fluorescence on the membrane reflected the progress of the proteolytic cleavage reaction.

The fluorescence of both GFP and Alexa Fluor 647-labeled TEV was monitored simultaneously. The average fluorescence intensity of each image was converted to the number of molecules on the surface using a calibration curve, enabling analysis of how the reaction rate and enzyme reactivity are modulated by the directionality of the environment. In this study, His-tagged GFP was injected at concentrations of 250 nM and 125 nM to control substrate density on the 2D membrane. Once the GFP substrate was anchored on the SLB, His-tagged TEV at 10 nM, 25 nM, and 50 nM was added to the sample to initiate the TEV proteolytic reaction. Images were captured at 5 s intervals, and the real-time TEV turnover rate was subsequently calculated.

Imaging Analysis

The mean fluorescence intensity of image stacks over the time course was analyzed using ImageJ's Plot Z-axis Profile function to quantify the change in GFP fluorescence on the membrane surface during the reaction (or the increase in fluorescence from Alexa Fluor 647-labeled TEV).

Single-molecule images of TEV were analyzed using the TrackMate plugin in ImageJ to count the number of TEV molecules recruited to the membrane. The LoG detector was applied to localize TEV molecules, with the estimated object diameter set to 0.75 μm. Approximately 400 to 500 molecules were detected per image.

Supplementary figures

A

Parameter set	0	1	2	3	4	5
Exposure time	50ms	50ms	20ms	20ms	20ms	20ms
Gain	3 (300)	1 (150)	1 (150)	1 (150)	1 (150)	1 (150)
power	50%	50%	15%	10%	5%	2%

Intensity ratio in parameter set	**Set 0 : Set 1**	1	0.153				
	**Set 1 : Set 2**		1	0.128			
	**Set 2 : Set 3**			1	0.678		
	**Set 3 : Set 4**				1	0.517	
	**Set 4 : Set 5**					1	0.406

Intensity ratio in parameter set	**Set 0 : Set 1**	1	0.153				
	**Set 0 : Set 2**	1		0.0196			
	**Set 0 : Set 3**	1			0.0133		
	**Set 0 : Set 4**	1				0.00686	
	**Set 0 : Set 5**	1					0.00279

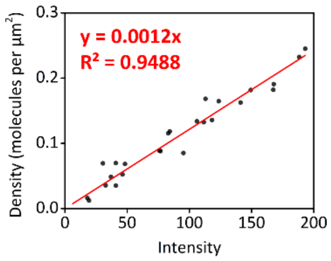

B

Parameter set	0	1	2	3	4	5
Exposure time	50ms	50ms	20ms	20ms	20ms	20ms
Gain	3 (300)	1 (150)	1 (150)	1 (150)	1 (150)	1 (150)
power	50%	50%	50%	25%	15%	10%

Intensity ratio in parameter set	**Set 0 : Set 1**	1	0.153				
	**Set 1 : Set 2**		1	0.413			
	**Set 2 : Set 3**			1	0.462		
	**Set 3 : Set 4**				1	0.571	
	**Set 4 : Set 5**					1	0.657

Intensity ratio in parameter set	**Set 0 : Set 1**	1	0.153				
	**Set 0 : Set 2**	1		0.0632			
	**Set 0 : Set 3**	1			0.0292		
	**Set 0 : Set 4**	1				0.0167	
	**Set 0 : Set 5**	1					0.0110

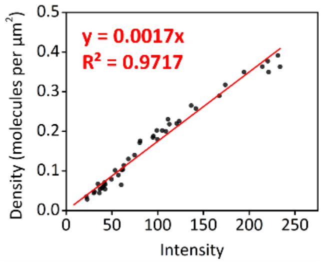

C

Parameter set	0	1	2	3	4
Exposure time	50ms	50ms	20ms	20ms	20ms
Gain	2 (300)	1 (300)	1 (300)	1 (300)	1 (300)
power	70%	20%	10%	5%	2%

Intensity ratio in parameter set	**Set 0 : Set 1**	1	0.072			
	**Set 1 : Set 2**		1	0.219		
	**Set 2 : Set 3**			1	0.503	
	**Set 3 : Set 4**				1	0.395

Intensity ratio in parameter set	**Set 0 : Set 1**	**1**	**0.072**			
	**Set 0 : Set 2**	**1**		**0.0158**		
	**Set 0 : Set 3**	**1**			**0.0079**	
	**Set 0 : Set 4**	**1**				**0.0031**

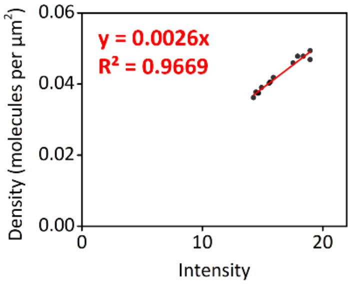

D

parameter	0	1	2	3	4
Exposure time	50ms	50ms	20ms	20ms	20ms
Gain	2 (300)	1 (150)	1 (150)	1 (150)	1 (150)
power	50%	20%	10%	5%	20%

Intensity ratio in parameter set	**Set 0 : Set 1**	1	0.098			
	**Set 1 : Set 2**		1	0.224		
	**Set 2 : Set 3**			1	0.493	
	**Set 2 : Set 4**			1		2.022

Intensity ratio in parameter set	**Set 0 : Set 1**	1	0.098			
	**Set 0 : Set 2**	1		0.0220		
	**Set 0 : Set 3**	1			0.0108	
	**Set 0 : Set 4**	1				0.0445

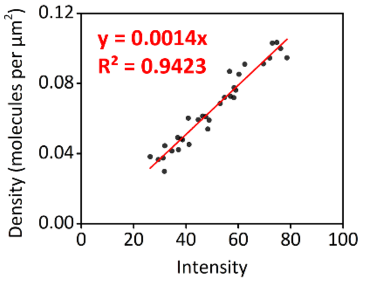

Figure S1. Calibration for determining the number of molecules on the SLB. (A) Calibration parameters for eGFP (table, left). A linear correlation is observed between the number of eGFP molecules on the SLB and the corresponding total fluorescence intensities from single-molecule images (plot, right). The number of eGFP molecules at the single-molecule level was quantified using ImageJ with the TrackMate plugin. Ratios of total fluorescence intensities from images acquired under two different EMCCD settings, at substrate densities at or above the single-molecule level, were used to calculate the molecular surface density per square micrometer on the SLB. Additional details are provided in the Results section. (B–D) Calibration parameters for His_7-TEV (B), mCherry (C), and His_4-TEV (D).

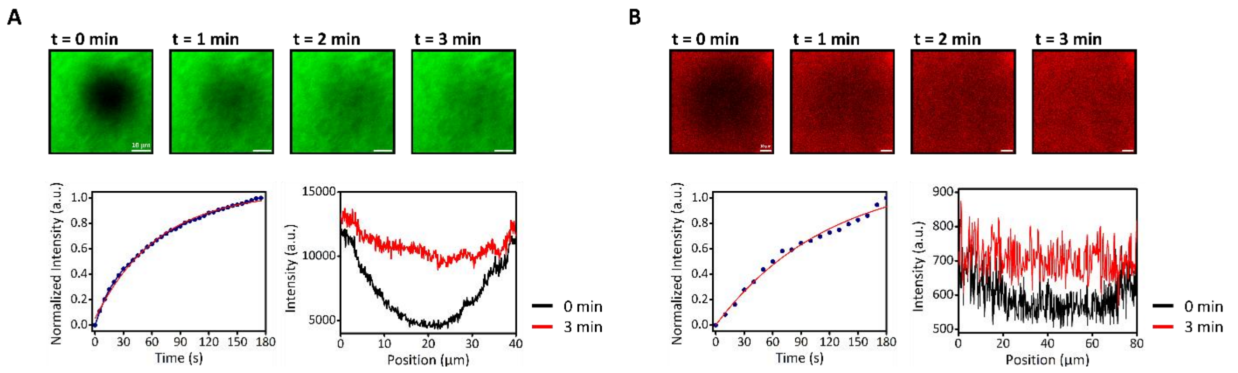

Figure S2. Fluorescence recovery after photobleaching (FRAP) of TEV substrates on the SLB. (A) FRAP analysis of eGFP on the SLB. After 1-minute exposure to high-power laser light, fluorescence images were acquired every 10 seconds to monitor recovery. The red solid line represents an exponential fit, yielding τ^D = 67 s and D = 1.92 μm²/s. (B) FRAP analysis of mCherry on the SLB. Similarly, fluorescence images were acquired every 10 seconds following 1-minute photobleaching. The red solid line represents an exponential fit with τ^D = 119 s and D = 1.08 μm²/s. These results indicate comparable diffusion coefficients for the GFP-tagged and mCherry-tagged substrates.

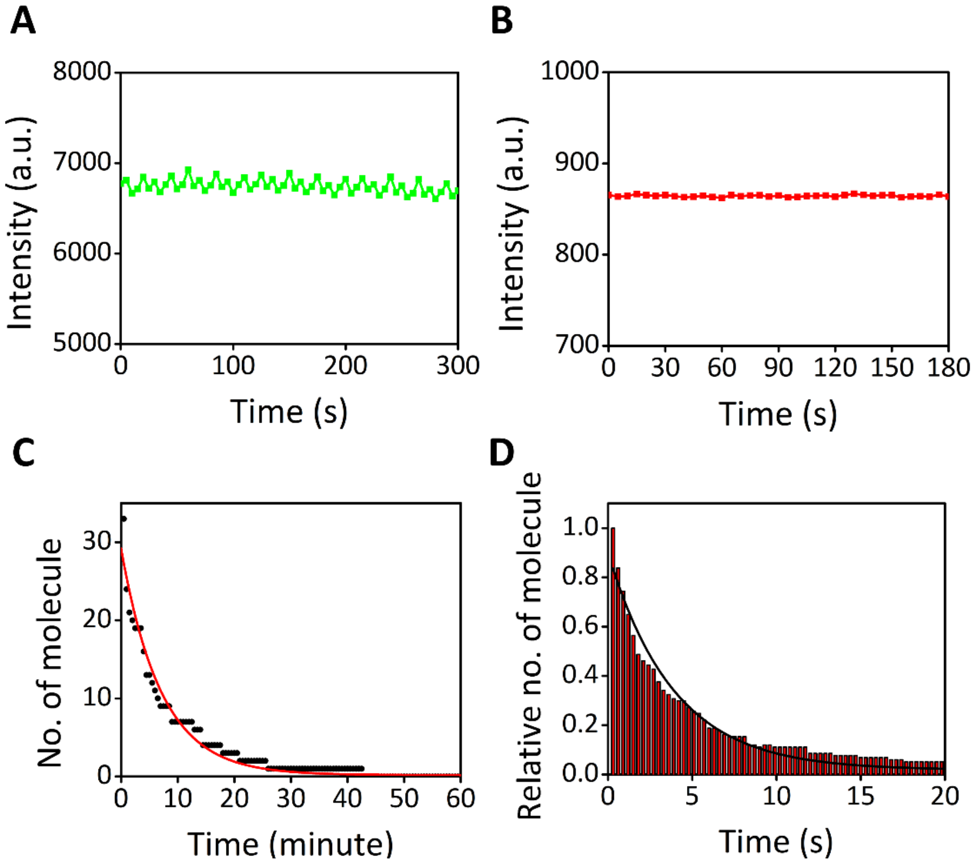

Figure S3. Control experiments evaluating photobleaching during fluorescence imaging. (A) Photobleaching curve of eGFP on the SLB. Images were acquired every 5 seconds with an exposure time of 20 ms and laser power set to 1.3 mW. No significant fluorescence loss was observed during time-lapse imaging. (B) Photobleaching curve of mCherry on the SLB (20 ms exposure, 1.7 mW laser power, one image every 5 seconds). No noticeable photobleaching was observed. (C) Cumulative histogram across time of fluorescent Alexa Fluor 647-labeled TEV anchored on a Ni^{2+}-coupled NTA-functionalized PLL-g-PEG surface. Images were acquired every 5 seconds (50 ms exposure, 15.6 mW laser power). The histogram was fitted with a single-exponential function (red line), yielding a time constant of 7.0 minutes, indicating that TEV fluorescence was not significantly affected by photobleaching under regular imaging conditions. (D) Cumulative histogram across time of fluorescent Alexa Fluor 647-labeled TEV under real-time fluorescence imaging. The protein was anchored on a Ni^{2+}-coupled NTA-functionalized PLL-g-PEG surface and imaged in no-delay mode (50 ms exposure, 15.6 mW laser power). The histogram was fitted with a single-exponential function (black line), yielding a time constant of 3.9 seconds.

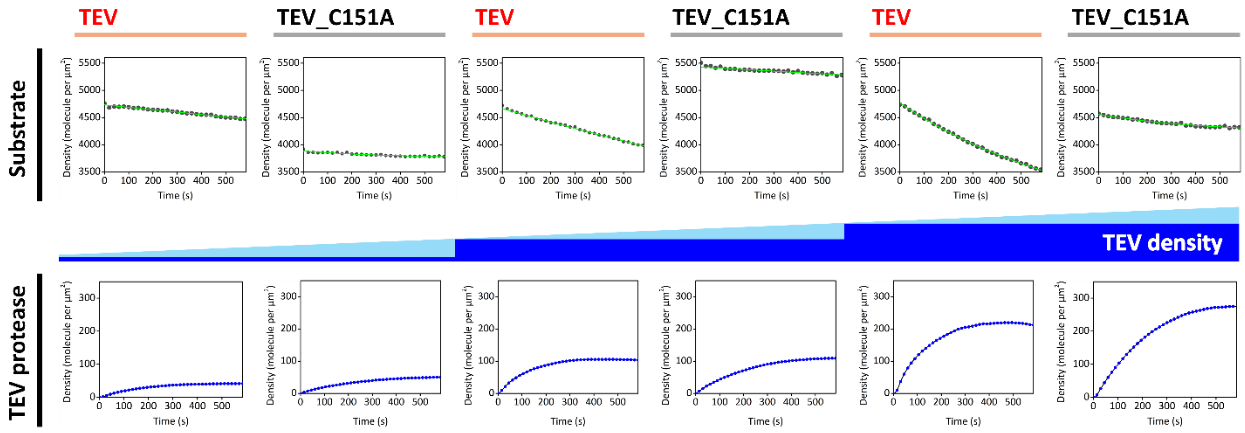

Figure S4. Basal dissociation of GFP substrate from the membrane measured using catalytically inactive TEV (TEV^{C151A}). The TEV mutant TEV^{C151A}, which lacks proteolytic activity (see Figure S5), was used to evaluate background dissociation of GFP substrate into the bulk solution. This dissociation results from unbinding between the His-tag and Ni^{2+}-NTA lipids, independent of enzymatic cleavage. Kinetic traces comparing the active and inactive TEV at surface densities of 50, 100, and 200 molecules per µm² show that TEV^{C151A} induces only a minimal decrease in GFP fluorescence. In contrast, the active TEV causes a substantial signal loss. These results confirm that GFP dissociation from the membrane is predominantly driven by the proteolytic cleavage reaction.

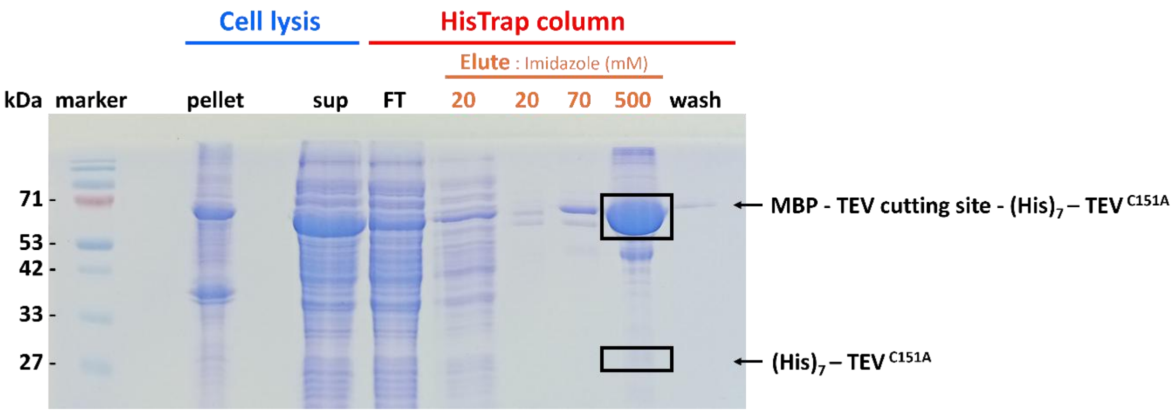

Figure S5. SDS-PAGE analysis of His7-TEV^{C151A} during protein expression and purification. A TEV cleavage site was inserted between MBP and His7-TEV to enable removal of the MBP fusion tag via autocleavage. In the case of the catalytically inactive mutant His7-TEV^{C151A}, the MBP tag remains uncleaved and is retained with the fusion protein during Ni^{2+}-NTA affinity purification. SDS-PAGE analysis shows a prominent band at 71 kDa, corresponding to the MBP–TEV^{C151A} fusion. No band is observed at 28 kDa (corresponding to TEV alone), confirming that TEV^{C151A} is catalytically inactive.

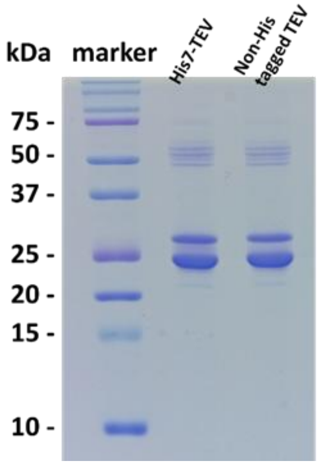

Figure S6. SDS-PAGE analysis comparing the enzymatic activity of His_7-TEV and non-His-tagged TEV proteases. This assay was performed to verify the consistency of enzymatic activity between His_7-TEV and non-His-tagged TEV, the latter of which is used in experiments where the enzyme is in solution and the substrate is membrane-bound. TEV protease and the substrate were mixed at a 1:50 (w/w) ratio, using 5.55 µg of TEV and 277 µg of substrate in a total volume of 40 µl. For convenience, the iron–sulfur cluster repair protein ScdA was used as the substrate. A TEV cleavage site was inserted between the His-tag and ScdA. The molecular weight of the His-tagged ScdA prior to cleavage is 27.92 kDa, and the cleaved ScdA product is 25.71 kDa. The reaction mixture was incubated at room temperature for 3 hours, and a 1 µl aliquot was analyzed by SDS-PAGE. Lane 1: protein ladder; Lane 2: cleavage product generated by His_7-TEV; Lane 3: cleavage product generated by non-His-tagged TEV. The gel shows comparable cleavage efficiency, indicating that His_7-TEV and non-His-tagged TEV exhibit similar enzymatic activity under the tested conditions.

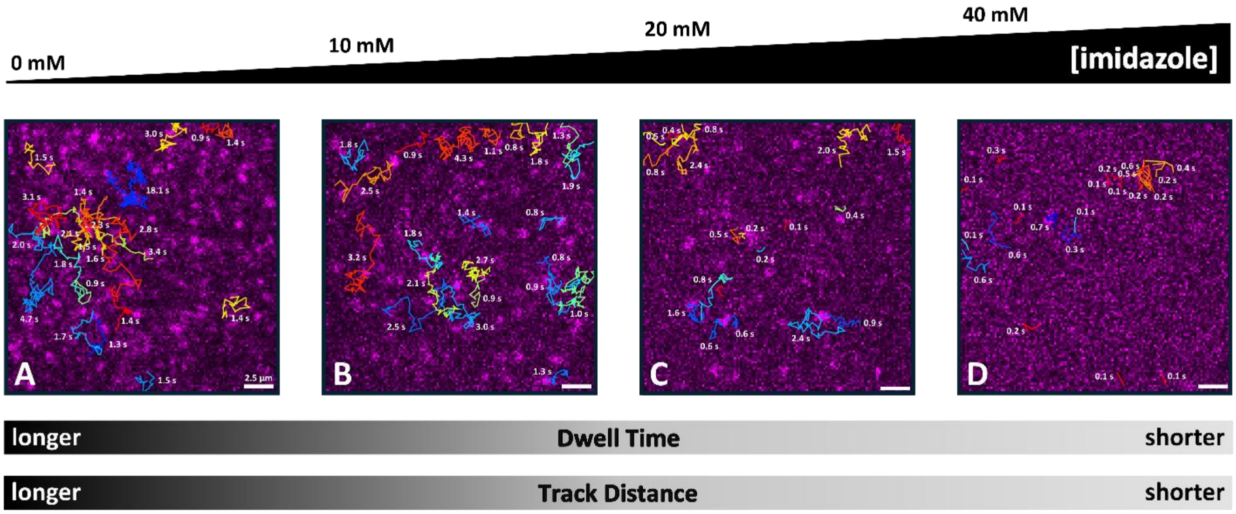

Figure S7. Single-molecule tracking analysis showing that the dwell time of His_4-TEV is modulated by imidazole concentration. Fluorescently labeled His_4-TEV (Alexa Fluor 647) was imaged by TIRF microscopy at imidazole concentrations of (A) 0 mM, (B) 10 mM, (C) 20 mM, and (D) 40 mM. Single-molecule trajectories were analyzed using the TrackMate plugin in ImageJ. To avoid overcrowding the figures, ~20 representative trajectories of TEV undergoing 2D Brownian motion on the membrane are shown in each panel (color-coded by track ID), with corresponding dwell times labeled near each track. The analysis of single-molecule dwell times (see Figure 5) reveals a clear trend: increasing imidazole concentrations result in shorter membrane dwell times for His_4-TEV.

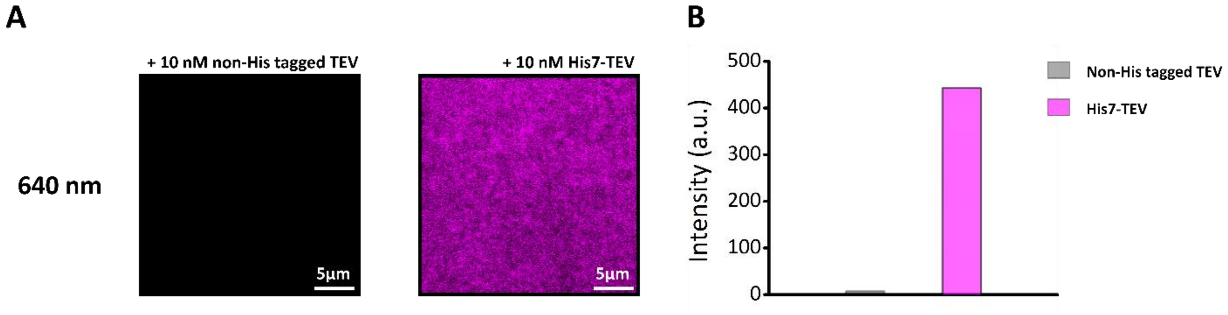

Figure S8. Recruitment of His-tagged TEV proteins to the SLB via His-tag chemistry. (A) Representative TIRF images comparing the recruitment of His_7-TEV and non-His-tagged TEV to the membrane. A 10 nM solution of Alexa Fluor 647-labeled TEV was introduced to an SLB containing 4% Ni^{2+}-NTA lipids, and images were acquired after 10 minutes of incubation. The injection of non-His-tagged TEV (left) shows negligible fluorescence under 640 nm excitation. In contrast, His-tagged TEV (right) yields a strong fluorescence signal due to specific binding via His-tag chemistry. The same brightness calibration was applied to both images. (B) Averaged fluorescence intensities of the images in (A), reflecting the signal from Alexa Fluor 647-labeled TEV.

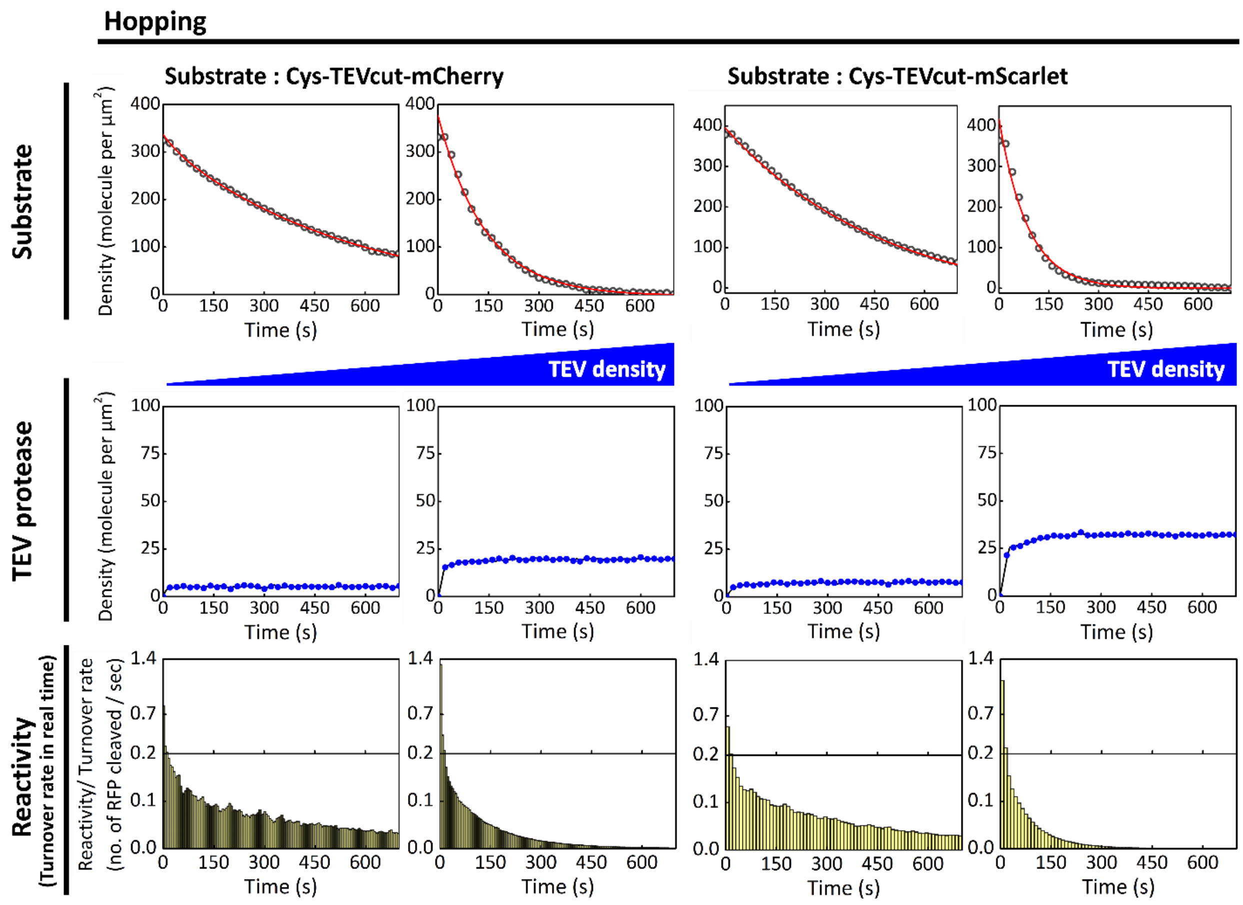

Figure S9. Validation of the hopping mechanism using mScarlet, a bright and stable variant of the red fluorescent protein mCherry used in Figure 6. The results obtained with mScarlet were consistent with those observed for mCherry. mScarlet was anchored to the membrane in the same configuration, with a TEV cleavage site positioned between an engineered N-terminal cysteine and the fluorophore. The surface density of mScarlet was maintained at ~350 molecules per µm² using maleimide conjugation chemistry. To induce the hopping mechanism, TEV protease with a His_4 tag was introduced in the presence of 40 mM imidazole, which weakens its interaction with the 4% Ni-NTA-containing SLB. This intermediate affinity allows TEV to dynamically bind, diffuse laterally, and dissociate from the membrane—facilitating a hopping-like movement across the membrane surface. Under these conditions, TEV molecules are repeatedly recruited to the membrane, enabling them to extend their effective reaction range and increase the probability of encountering substrates. This dynamic behavior reduces the likelihood of local substrate depletion and helps maintain a relatively high catalytic turnover rate. The equilibrium in membrane-associated TEV density reflects the balance between recruitment and dissociation. Similar to the observations with mCherry, higher TEV densities led to a faster decline in turnover due to accelerated substrate consumption.

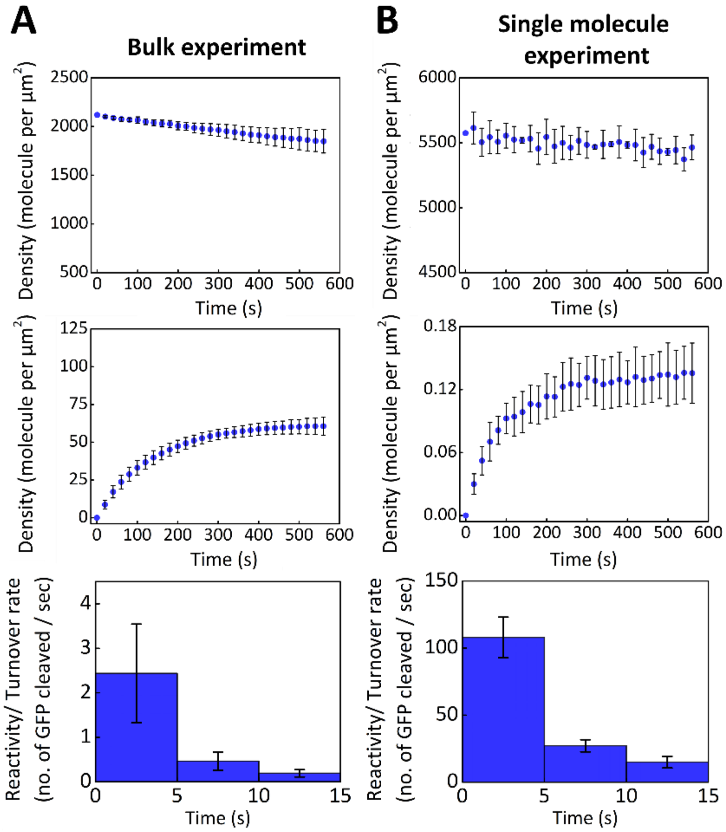

Figure S10. Statistical analysis of experimental variability for the trials shown in Figures 3 and 4. (A) For the experiment corresponding to Figure 3A, the surface density of GFP-tagged substrate on the SLB was controlled at approximately 2000 molecules per μm². After anchoring the substrate, 25 nM His-tagged TEV protease was added to initiate the cleavage reaction. Images were acquired at 5-second intervals, and real-time TEV turnover rates were calculated at t = 5, 10 and 15 seconds. (B) In the experiment corresponding to Figure 4, the surface density of GFP-tagged substrate was adjusted to approximately 5000 molecules per μm². TEV recruitment was tuned such that ~400–500 molecules (corresponding to ~0.12 molecules per μm²) were present within the imaging field. The real-time turnover rates were again calculated at t = 5, 10 and 15 seconds.

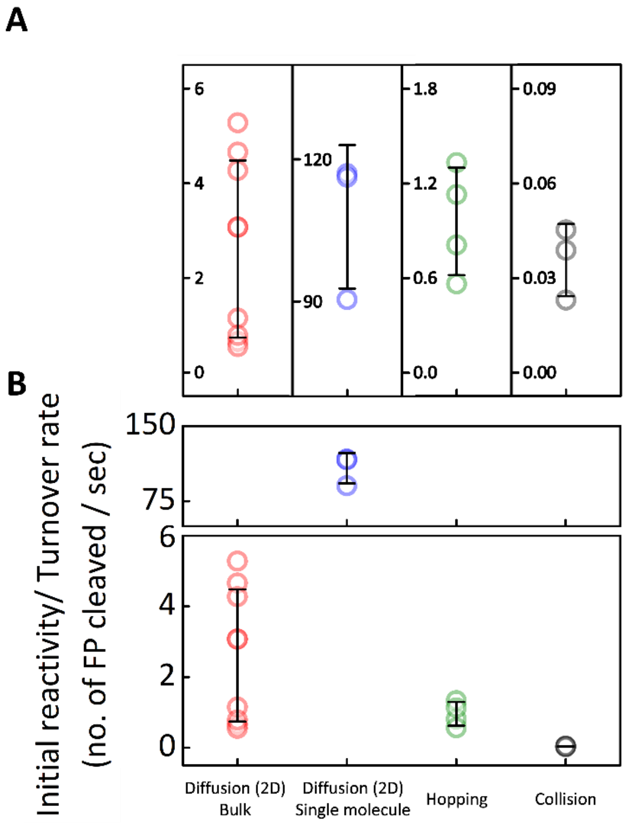

Figure S11. Statistical analysis of experimental variability in the initial turnover rates across different experimental scenarios. (A) Zoom-in view of the individual initial turnover rates for each scenario. (B) Combined plot of initial turnover rates. A visual break in the y-axis is introduced between 6 and 60 substrate molecules cleaved per second to accommodate the wide dynamic range of the data. Above the break, the y-axis scale is adjusted such that one division corresponds to 75 substrate molecules cleaved per second.
